# Multimorbidity in the working-age population in Brazil: sociodemographic and behavioral factors

**DOI:** 10.11606/s1518-8787.2026060007251

**Published:** 2026-08-07

**Authors:** Tamyres Araújo Andrade Donato, Amanda Cristina de Souza Andrade, Vanessa Moraes Bezerra

**Affiliations:** I Universidade Federal da Bahia Instituto Multidisciplinar em Saúde Vitória da Conquista BA Brasil Universidade Federal da Bahia. Instituto Multidisciplinar em Saúde. Vitória da Conquista, BA, Brasil; II Universidade Estadual do Sudoeste da Bahia Departamento de Ciências da Saúde Vitória da Conquista BA Brasil Universidade Estadual do Sudoeste da Bahia. Departamento de Ciências da Saúde. Vitória da Conquista, BA, Brasil; III Fundação Oswaldo Cruz Instituto René Rachou Belo Horizonte MG Brasil Fundação Oswaldo Cruz. Instituto René Rachou. Belo Horizonte, MG, Brasil; VI Universidade Federal do Mato Grosso Instituto de Saúde Coletiva Programa de Pós-Graduação em Saúde Coletiva Cuiabá MT Brasil Universidade Federal do Mato Grosso. Instituto de Saúde Coletiva. Programa de Pós-Graduação em Saúde Coletiva. Cuiabá, MT, Brasil

**Keywords:** Multimorbidity, Economically Active Population, Brazil, Health Survey, Epidemiological Studies

## Abstract

**OBJECTIVE:**

To assess the prevalence of multimorbidity in the Brazilian working-age population and analyze its determinants, with an emphasis on sociodemographic and behavioral aspects.

**METHODS:**

A cross-sectional study was conducted using data from the 2019 National Survey of Health, involving employed individuals aged 18 years or older. The outcome was multimorbidity, characterized by the simultaneous presence of two or more chronic noncommunicable diseases, based on a list of 14 conditions. Poisson regression with robust variance was used to assess the association between sociodemographic variables, habits, behaviors, and health/service outcomes.

**RESULTS:**

Among the 52,475 employed individuals, 22.6% (95%CI 21.9–23.2) had multimorbidity, which was more prevalent among: women; those aged 30 or older; those with lower levels of education; those who self-identified as white; and urban residents. Participation in public programs for physical activity, episodic heavy drinking, leisure-time screen time, self-rated health as fair/poor or very poor, a doctor’s visit in the past year, and having health insurance were also positively associated with multimorbidity.

**CONCLUSION:**

The findings highlight the significant prevalence of multimorbidity among the working-age population in Brazil, with particular emphasis on social and behavioral determinants. These results reinforce the importance of health promotion strategies in multiple contexts, including workplaces, communities, and primary care settings.

## INTRODUCTION

Chronic noncommunicable diseases (CNCDs) primarily affect populations that are more exposed to risk factors, such as socioeconomic and lifestyle factors, or that have limited access to health information and services. The increasing prevalence of CNCDs and their coexistence in a single individual result in a poorer quality of life, limitations, disabilities, and high premature mortality^1^, constituting a public health problem.

Multimorbidity is defined as the coexistence of two or more chronic diseases in a single individual^1^. Prevalence estimates vary widely depending on the context and the criteria used. In an international study of older adults, the prevalence was 55.1%^2^. In Brazil, a population-based study of adults aged 20 to 59 years estimated a prevalence of 10.9%, based on self-reported medical diagnoses^3^. Among the working population, marked heterogeneity is observed: a prevalence of 0.9% among European workers^1^ and higher rates in Brazil, ranging from 28.6% among urban workers to 41.5% among rural workers^4,5^. These findings highlight the influence of the occupational context and social inequalities on the occurrence of multimorbidity.

Currently, multimorbidity stands out as a major cause of disability among working individuals, increasing the risk of workplace accidents, reducing work performance, and increasing absenteeism^5^. In addition to physical impairments, multimorbidity increases the demand for health care services, including medical visits, hospitalizations, and emergency services. This scenario can have significant economic impacts due to reduced productive capacity, prolonged absences, and increased use of healthcare and social security services^6^.

Several factors experienced by the working population influence the development of multimorbidity, including job instability, low wages, multiple employment relationships, and productivity pressures, which act as chronic stressors^6^. In addition to stress, work itself and the work environment can constitute health risk factors, such as physical, chemical, and ergonomic exposures, which have already been linked to cardiovascular, musculoskeletal, and neoplastic conditions^4,5^. Furthermore, long workdays, shift work, lengthy commutes, and rigid routines make it difficult to adopt healthy behaviors, such as regular physical activity^5^. These conditions also limit timely access to health services, compromising efforts in health promotion, prevention, early diagnosis, and monitoring of chronic conditions^7^.

Considering this scenario, the Ministry of Health launched, in 2021, the Strategic Action Plan to Tackle NCDs (2021–2030), which proposes preventing them through changes in production processes, health promotion strategies, and interventions targeting occupational risk factors^8^. Research on multimorbidity among employed individuals in Brazil can generate evidence to support policymakers, health professionals, and employers in formulating policies that contribute to its prevention^5^.

Studies using data from the National Survey of Health (PNS) have investigated multimorbidity in different segments of the Brazilian population, including the general population^9^, older adults^10^, young people^11^, as well as specific occupational subgroups, such as agricultural workers^7^. However, few have specifically addressed multimorbidity among employed individuals, who represent the country’s economically active and productive population. Given that multimorbidity can arise early, especially in contexts of social inequality^3^, it is necessary to understand the associated factors in this population to inform health promotion, prevention, and intersectoral planning policies.

This study aimed to assess the prevalence of multimorbidity in the Brazilian employed population and to analyze the associated sociodemographic and behavioral factors.

## METHODS

### Study Design

A cross-sectional study using data from the PNS, a national household-based epidemiological survey conducted in 2019 by the *Instituto Brasileiro de Geografia e Estatística* (IBGE - Brazilian Institute of Geography and Statistics) in partnership with the Ministry of Health. The information, which was collected through household interviews, included socioeconomic characteristics, the presence of diseases, and lifestyle factors^12^. A total of 94,114 households were visited, and 90,846 interviews were conducted^13^. Of these, 52,475 individuals aged 18 years or older reported being employed or working during the reference week (July 21 to 27, 2019)^14^, comprising the sample for this study.

### Outcome

The outcome was multimorbidity, defined as the simultaneous presence of two or more CNCDs^3,11^, assessed based on 14 conditions: hypertension, diabetes, hypercholesterolemia, heart conditions (heart attack, angina, or heart failure), stroke, asthma, arthritis or rheumatism, chronic spinal conditions (chronic back or neck pain, low back pain, sciatica, vertebral or disc conditions), work-related musculoskeletal disorders, depression, other mental disorders (anxiety disorder, panic disorder, schizophrenia, bipolar disorder, psychosis, or obsessive-compulsive disorder), lung diseases (emphysema, chronic bronchitis, or chronic obstructive pulmonary disease), cancer, and chronic kidney disease. These were identified using the question: “Has a doctor ever diagnosed you with (name of the disease)?”^12^. The multimorbidity variable was dichotomized as follows: “Yes,” indicating the presence of two or more CNCDs; “No,” indicating the absence of CNCDs or the presence of only one CNCD^10^.

### Independent Variables

The independent variables were selected based on a prior review of the literature^10,11,15,16^ and were divided into the following categories: sociodemographic factors, habits and behaviors, and health/service. The sociodemographic category included: sex (male, female); age (18–29, 30–39, 40–59, ≥ 60 years); educational attainment (no schooling to incomplete high school, complete high school to complete higher education); self-reported race/ethnicity (white, Black/Brown). The proportion of individuals who self-identified as Indigenous (702; 0.75%) and Asian (692; 0.74%) was low in the PNS sample. Given the small number of observations and the possibility of imprecise estimates, we chose to restrict the analysis to the white and Black/Brown categories. 24-hour shift work (yes, no); night work (yes, no); and place of residence (urban, rural).

In the habits and behaviors section, the following were considered: participation in a public program for physical activity (yes, not familiar with it/do not participate); episodic heavy drinking (consumption of five or more alcoholic drinks on a single occasion for both men and women); current smoking (yes, no); physical activity (those who engaged in less than 150 minutes per week of physical activity—in the domains of leisure, work, and commuting to work—were classified as inactive)^15^. Group physical activity (sports, exercise, recreational, or artistic activities) was dichotomized into “more than once a week to three times a month” and “Does not participate/Only a few times a year.” Those who spent 3 hours or more per day watching TV or using a computer, tablet, or cell phone for leisure (excluding work) were classified as having excessive screen time^16^.

In the health/service section, the following were considered: self-assessed health (very good/good, fair/poor/very poor)^10^; a doctor’s visit in the past year (yes: within the past year, no: more than a year ago/never)^11^; and health insurance coverage (yes, no)^10^.

### Statistical Analysis

A descriptive analysis was performed, and the prevalence of multimorbidity and the 95% confidence interval (95%CI) were estimated. To identify associated factors, bivariate and multivariate analyses were conducted, with estimates of prevalence ratios (PR) and 95%CIs calculated using Poisson regression with robust variance. The multivariate analysis followed a hierarchical block-wise variable entry strategy^17^. Initially, a bivariate analysis was performed, and variables with a p-value < 0.20 were eligible for inclusion in the multivariate analysis. This criterion was adopted to avoid the premature exclusion of potential confounding factors. In the multiple analysis with hierarchical entry, variables were entered in blocks according to the proposed model (Figure). For each block (sociodemographic, habits and behaviors, and health/service), a multiple model was fitted, successively excluding the variables with the highest p-values until only those with a p-value ≤ 0.05 remained in each block after adjustment for variables within the same block and from hierarchically higher blocks.

**Figure:**
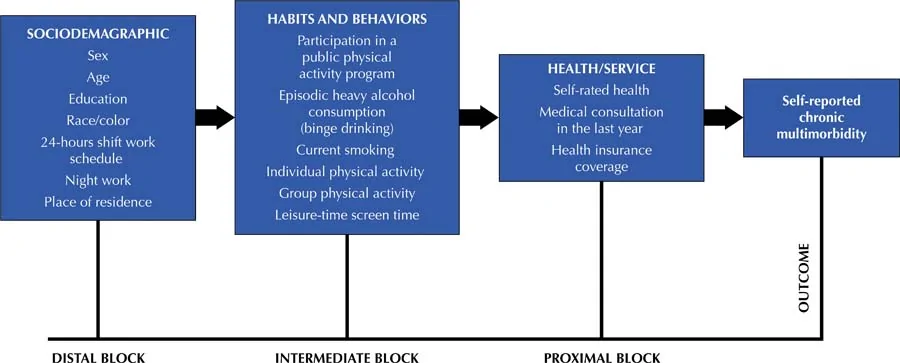
Hierarchical model for data analysis.

The Akaike information criterion (AIC) was used to compare models, with the model having the lowest AIC considered the best. The analyses were performed in Stata (version 16), in survey mode, which accounts for the complex sample design.

### Ethical Considerations

The PNS was approved by the National Research Ethics Commission (opinion No. 3,529,376).

## RESULTS

Of the 52,475 employed individuals, 11,638 (22.6%; 95%CI 21.9–23.2) had multimorbidity. The most prevalent CNCDs (Table 1) were hypertension (19.3%), chronic spinal problems (19.0%), hypercholesterolemia (13.2%), depression (7.7%), and diabetes (5.4%).

**Table 1:** Prevalence of self-reported chronic noncommunicable diseases in the Brazilian working-age population, National Health Survey, 2019 (n = 52,475).

Self-reported chronic noncommunicable diseases	Prevalence (%)	95%CI
Hypertension	19.3	19.0–19.7
Chronic spinal problem	19.0	18.6–19.3
Hypercholesterolemia	13.2	12.8–13.4
Depression	7.7	7.4–7.9
Diabetes mellitus	5.4	5.2–5.6
Other mental disorders	5.1	4.9–5.2
Arthritis or rheumatism	4.9	4.8–5.1
Asthma	4.8	4.6–5.0
Cardiovascular diseases	3.2	3.0–3.3
Work-related rheumatic diseases	2.2	2.1–2.4
Cancer	1.6	1.5–1.7
Chronic kidney disease	1.1	1.0–1.2
Stroke	1.0	0.9–1.1
Lung diseases	1.0	0.9–1.1
Multimorbidity	22.6	21.9–23.2

95%CI: 95% confidence interval.

Regarding the characteristics of the sample (Table 2), a higher percentage of men (55.5%), individuals aged 40 to 59 years (41.1%), those with an educational level ranging from complete high school to complete higher education (59.8%), and those of Black or mixed race (55.3%) were observed. Regarding occupational characteristics, 1.7% worked 24-hour shifts and 13.5% worked night shifts. A higher percentage of participants resided in urban areas (87.7%). Regarding habits and behaviors, the majority did not participate in public programs for physical activity; 22.1% reported consuming five or more alcoholic drinks on a single occasion, in the past 30 days; 12.9% used tobacco; 27.6% were physically inactive; 59.5% reported not engaging in group physical activities or doing so only a few times in the past year; and 35% spent three or more hours a day sitting in front of screens. Regarding health variables, 26.2% rated their health as fair, poor, or very poor; 21.9% had not seen a doctor in the past year; and 69.7% did not have health insurance.

**Table 2:** Characteristics of the Brazilian working population, National Survey of Health, 2019 (n = 52,475).

Variables	Prevalence (%)	95%CI
**Sociodemographics**		
Sex		
Male	55.5	54.7–56.4
Female	44.5	43.6–45.3
Age		
18–29 years	23.2	22.4–24.0
30–39 years	26.9	26.3–27.7
40–59 years	41.1	40.3–41.8
≥ 60 years	8.8	8.4–9.2
Education		
Complete high school to complete higher education	59.8	59.0–60.7
No education to incomplete high school education	40.2	39.3–41.0
Race/Ethnicity		
Black/Brown	55.3	54.4–56.2
White	44.7	43.8–45.6
24-hour shift work		
Yes	1.7	1.5–1.9
No	98.3	98.2–98.5
Night work		
Yes	13.5	12.9–14.1
No	86.5	85.9–87.1
Place of residence		
Rural	12.3	11.9–12.8
Urban	87.7	87.2–88.1
**Habits and behaviors**		
Participation in a public program for physical activity		
Does not participate/is not aware of it	97.7	97.4–97.9
Yes	2.3	2.1–2.6
Episodic heavy drinking		
Yes	22.1	21.4–22.8
No	77.9	77.2–78.6
Current tobacco use		
Yes	12.9	12.4–13.5
No	87.1	86.5–87.6
Physical activity		
Active	72.4	71.6–73.2
Inactive	27.6	26.8–28.3
Group physical activity in the past year		
> Once a week to three times a month	40.5	39.7–41.4
Does not participate/only a few times a year	59.5	59.5–58.6
Leisure screen time		
≥ 3 hours	35.0	34.1–35.8
< 3 hours	65.0	64.2–65.9
**Health/service**		
Self-assessed health		
Very good/good	73.8	73.2–74.5
Fair/poor/very poor	26.2	25.5–26.9
Doctor’s visit in the past year		
No	21.9	21.3–22.5
Yes	78.1	77.5–78.8
Health insurance coverage		
No	69.7	68.7–70.6
Yes	30.3	29.4–31.2

95%CI: 95% confidence interval.

In the bivariate analysis, multimorbidity was statistically significantly associated with sociodemographic, behavioral, and health-related variables, including sex, age, education level, race/ethnicity, place of residence, participation in a public program for physical activity, episodic heavy drinking, physical activity, group physical activity, leisure-time screen time, self-rated health, doctor visits in the past year, and health insurance coverage (Table 3). The observed associations varied in direction and magnitude and were further explored in the subsequent adjusted analysis.

**Table 3:** Prevalence and crude prevalence ratio of multimorbidity according to sociodemographic characteristics, habits and behaviors, and health/service conditions in the Brazilian working-age population, National Survey of Health, 2019.

Variables	Prevalence (%) (95%CI)	PR (95%CI)	p-value
**Sociodemographics**			
Sex			
Male	17.4 (16.6–18.2)	1.00	< 0.001
Female	29.0 (27.9–30.1)	1.67 (1.57–1.76)	
Age			
18–29 years	8.9 (7.9–9.9)	1.00	< 0.001
30–39 years	14.7 (13.7–15.8)	1.66 (1.45–1.90)	
40–59 years	30.2 (29.1–31.3)	3.41 (3.03–3.83)	
≥ 60 years	46.8 (44.5–49.1)	5.28 (4.68–5.96)	
Education			
Complete high school to complete higher education	20.6 (19.7–21.5)	1.00	< 0.001
No education to incomplete high school education	25.5 (24.4–26.6)	1.23 (1.17–1.31)	
Race/Ethnicity			
Black/Brown	20.9 (20.0–21.7)	1.00	< 0.001
White	24.6 (23.6–25.7)	1.18 (1.12–1.25)	
24-hour shift work			
Yes	19.0 (15.5–23.0)	1.00	0.083
No	22.6 (21.9–23.2)	1.19 (0.98–1.45)	
Night work			
Yes	22.2 (20.5–24.0)	1.00	0.691
No	22.6 (21.9–23.3)	1.01 (0.93–1.11)	
Place of residence			
Rural	19.6 (18.4–20.9)	1.00	< 0.001
Urban	23.0 (22.2–23.7)	1.17 (1.08–1.26)	
**Habits and behaviors**			
Participation in a public program for physical activity			
Does not participate/is not aware of it	22.3 (21.7–23.0)	1.00	< 0.001
Yes	31.5 (26.9–36.5)	1.41 (1.20–1.64)	
Episodic heavy drinking			
No	23.6 (22.8–24.4)	1.00	< 0.001
Yes	19.0 (17.9–20.2)	0.80 (0.75–0.86)	
Current tobacco use			
No	22.6 (21.8–23.3)	1.00	0.863
Yes	22.4 (20.7–24.2)	0.99 (0.91–1.07)	
Physical activity			
Active	21.6 (20.8–22.3)	1.00	< 0.001
Inactive	25.1 (23.9–26.4)	1.17 (1.09–1.24)	
Group physical activity in the past year			
> Once a week to three times a month	19.8 (18.8–20.9)	1.00	< 0.001
Does not participate/only a few times a year	24.4 (23.6–25.3)	1.23 (1.16–1.31)	
Leisure screen time			
< 3 hours	23.3 (22.4–24.1)	1.00	0.002
≥ 3 hours	21.2 (20.2–22.3)	0.91 (0.86–0.96)	
**Health/service**			
Self-assessed health			
Very good/good	16.0 (15.3–16.7)	1.00	< 0.001
Fair/poor/very poor	41.1 (39.7–42.5)	2.57 (2.44–2.71)	
Doctor’s visit in the past year			
No	8.0 (7.2–8.8)	1.00	< 0.001
Yes	26.6 (25.8–27.5)	3.34 (3.01–3.70)	
Health insurance coverage			
No	20.8 (20.1–21.5)	1.00	< 0.001
Yes	26.6 (25.3–28.0)	1.28 (1.20–1.36)	

PR: prevalence ratio; 95%CI: 95% confidence interval.

In the multiple Poisson regression analysis (Table 4), the prevalence of multimorbidity was higher among women (PR = 1.67; 95%CI 1.59–1.77), and showed a dose-response gradient with age; that is, the older the age, the higher the prevalence of multimorbidity (OR = 5.14; 95%CI 4.54–5.83). Employed individuals with lower educational attainment (OR = 1.08; 95%CI 1.02–1.15), white individuals (OR = 1.11; 95%CI 1.05–1.17), and urban residents (OR = 1.11; 95%CI 1.04–1.19) had a higher prevalence of the outcome. Participation in public programs for physical activity (PR = 1.26; 95%CI 1.09–1.44), episodic heavy drinking (PR = 1.08; 95%CI 1.01–1.15), and leisure-time screen time (PR = 1.13; 95%CI 1.07–1.19). For health variables, a positive association with multimorbidity was found for negative self-rated health (OR = 2.12; 95%CI 2.01–2.23), a doctor’s visit in the past year (OR = 2.45; 95%CI 2.20–2.72), and having health insurance (OR = 1.26; 95%CI 1.19–1.33).

**Table 4:** Multiple Poisson regression analysis of factors associated with multimorbidity in the Brazilian working-age population, National Survey of Health, 2019.

Variables	Model 1^a^		Model 2^b^		Model 3^c^	
	OR	95%CI	OR	95%CI	PR	95%CI
**Sociodemographics**						
Gender						
Male	1.00	-	1.00	-	1.00	-
Women’s	1.67	1.59–1.77	1.69	1.60–1.79	1.48	1.40–1.56
Age						
18–29 years	1.00	-	1.00	-	1.00	-
30–39 years	1.65	1.45–1.89	1.70	1.48–1.94	1.60	1.40–1.82
40–59 years	3.30	2.93–3.73	3.44	3.05–3.87	2.91	2.59–3.28
≥ 60 years	5.14	4.54–5.83	5.36	4.73–6.08	4.09	3.61–4.64
Education						
Complete high school to complete higher education	1.00	-	1.00	-	1.00	-
No education to incomplete high school education	1.08	1.02–1.15	1.09	1.03–1.16	1.06	0.99–1.13
Race/Ethnicity						
Black/Brown	1.00	-	1.00	-	1.00	-
White	1.11	1.05–1.17	1.11	1.06–1.18	1.15	1.09–1.21
Place of residence						
Rural	1.00	-	1.00	-	1.00	-
Urban	1.11	1.04–1.19	1.08	1.01–1.16	1.05	0.98–1.12
**Habits and behaviors**						
Participation in a public program for physical activity						
Does not participate/is not aware of it			1.00	-	1.00	-
Yes			1.26	1.09–1.44	1.26	1.11–1.44
Episodic heavy drinking						
No			1.00	-	1.00	-
Yes			1.08	1.01–1.15	1.11	1.04–1.19
Leisure screen time						
< 3 hours			1.00	-	1.00	-
≥ 3 hours			1.13	1.07–1.19	1.13	1.07–1.19
**Health/service**						
Self-assessed health						
Very good/good					1.00	-
Fair/poor/very poor					2.12	2.01–2.23
Doctor’s visit in the past year						
> More than a year ago or never					1.00	-
Up to one year					2.45	2.20–2.72
Health insurance coverage						
No					1.00	-
Yes					1.26	1.19–1.33
Akaike Criterion^d^	4025063		4021596		3801403	

PR: prevalence ratio; 95%CI: 95% confidence interval.

^a^ Model 1: adjusted for sociodemographic variables.

^b^ Model 2: adjusted for sociodemographic variables and habits and behaviors.

^c^ Model 3: adjusted for sociodemographic variables, habits and behaviors, and health/service.

^d^ Weighted by sample weight.

## DISCUSSION

This study revealed a high prevalence of multimorbidity in the Brazilian working-age population, especially among women and older adults. An association was also observed with socioeconomic and contextual conditions, such as lower educational attainment and residence in urban areas. Furthermore, multimorbidity was related to risk behaviors and greater use of health services, suggesting that its occurrence results from the interaction between sociodemographic, behavioral, and access-to-care factors.

The most prevalent conditions in this study, such as hypertension, chronic spinal problems, hypercholesterolemia, depression, diabetes, and other mental disorders, are consistent with the multimorbidity profile described in the literature for the general population and among workers^3,6,11^. Currently, in Brazil, musculoskeletal and connective tissue disorders (ICD-10 M) and mental and behavioral disorders (ICD-10 F) rank among the leading causes of work-related sick leave, whether due to accidents or non-accidents^18^. These conditions are strongly associated with adverse occupational environments, characterized by long working hours, job instability, and high pressure to be productive, which reinforces the role of work in triggering and exacerbating chronic conditions with physical and mental repercussions^5^. As a result, these conditions negatively impact productivity, leading to increased absenteeism, disability, and early retirement, in addition to placing a significant burden on the health care and social security systems^1^.

In this study, the prevalence of multimorbidity was higher among women than among men, a finding already well established in the literature^5,9^. Women’s greater use of health care services allows for greater access to medical diagnoses^9^. Moreover, the dual burden of balancing professional work with domestic and family responsibilities increases exposure to stress and may contribute to the development of chronic diseases^19^.

Multimorbidity was more prevalent among employed individuals aged 30 years or older, indicating the presence of multiple chronic conditions in economically active groups. The working-age population is exposed to health-risk behaviors and various stressful situations, which contribute to the accumulation of diseases over time^6,19^. This finding reinforces the originality of the analysis by demonstrating that chronic diseases can emerge early, possibly triggered by social contexts and lifestyle habits, even before a long occupational career^3,11^. This early onset of illness has implications for both health and the economy, as individuals of working age with multimorbidity may face barriers to entering and remaining in the labor market, as well as a higher risk of layoffs, early retirement, and receipt of disability benefits^1,6^. Specific policies and programs^7^ are needed for these vulnerable groups to reduce multimorbidity, absenteeism, and costs associated with CNCDs^1^. Prevention and health care for the working population should be viewed as strategic investments in economic development.

Lower educational attainment was positively associated with multimorbidity, a finding also observed in other population groups^1^. Educational attainment reflects socioeconomic status and influences occupational trajectories, exposure to poor working conditions, and access to health protection resources. Contexts of vulnerability—characterized by unstable employment, psychosocial stress, and limited access to health services—contribute to the early onset and inadequate management of chronic conditions^20,21^. Furthermore, housing conditions constitute an important social determinant of health. Populations living in socially neglected urban peripheries face greater socioeconomic deprivation, reduced access to urban infrastructure and public services, and greater exposure to adverse environmental factors, contributing to the worsening of chronic diseases and the occurrence of multimorbidity at earlier ages^20,21^.

In this study, a higher prevalence of multimorbidity was observed among white individuals. However, it is important to note that this outcome was based on self-reported medical diagnoses, which may reflect differences in access to and utilization of health care services. White individuals generally have higher levels of education and income and, consequently, are more likely to receive a timely diagnosis of chronic diseases, which may overestimate the prevalence in this group when compared to the Black population^22^. Recent evidence, both international and national, has demonstrated a higher burden of multimorbidity among Black and Brown individuals, especially when structural markers of inequality are taken into account^22,23^. These inequalities develop over the course of a lifetime and include greater exposure to adverse socioeconomic conditions, spatial segregation, and experiences of racial discrimination, factors associated with cumulative chronic disease burden^23^. Thus, studies based exclusively on self-reported diagnoses may underestimate racial health inequities, reinforcing the need for analyses that incorporate structural and contextual determinants in understanding multimorbidity^22^.

Employed individuals living in urban areas had a higher prevalence of multimorbidity, a finding similar to that reported in a study conducted in Colombia^23^. The greater availability of health services in these areas facilitates the diagnosis of diseases^10,19^. Urban environments also expose individuals to stressors such as traffic, violence, and pollution, which may contribute to multimorbidity^7^. In rural areas, however, geographic barriers, poor transportation, and higher levels of poverty hinder access to services, resulting in fewer opportunities for care^7^. In this study, since diagnoses were self-reported, those with greater access to health services were more likely to report diseases.

Regarding habits and behaviors, participation in public programs promoting physical activity was higher among employed individuals with multimorbidity. People with CNCDs use health services more frequently and receive more information about these initiatives^24^. Access to public spaces that encourage healthy habits is essential for preventing CNCDs by reducing health-risk behaviors, which in turn promotes a more active lifestyle^11^. The Ministry of Health’s strategy for controlling these diseases prioritizes active aging^8^. Since many individuals of working age face barriers to regular physical activity, it is important that public activities be accessible and offered at flexible times^24^. Interventions are also needed in health policies and workplace environments to promote light, short-duration activities—such as walking and using stairs—incorporated into the work routine^25^.

In this study, employed individuals who reported consuming five or more drinks on a single occasion in the past thirty days had a higher prevalence of multimorbidity. This finding is particularly relevant among younger individuals, given that excessive alcohol consumption can accelerate the onset of chronic diseases during working years, which underscores the need for early interventions^26^. Since this is a modifiable risk factor, it is essential to invest in public policies for prevention and intervention specifically tailored to this population.

Episodic heavy drinking may be related to specific work conditions, such as long work hours, job insecurity, undervalued work, and high levels of physical or mental exertion^26^. Contextual, sociodemographic, and lifestyle factors may also contribute to this behavior^27^. This pattern of consumption negatively affects the occupational, family, and social spheres^26^. As coping strategies, it is recommended to expand and improve the screening and diagnosis of excessive alcohol consumption at work and during leisure time, ensuring access to psychosocial rehabilitation services when necessary^28^. Additionally, intersectoral actions involving regulatory and health agencies are essential to reduce consumption, prioritizing vulnerable groups^27^.

Screen time, excluding time spent sitting at work, was positively associated with multimorbidity, especially among young working adults (ages 18–39). The increase in time spent on passive leisure activities, such as screen use, may explain this finding^29^. Excessive working hours and the type of work performed also promote sedentary behavior^30^, which contributes to other risks and can have negative impacts on physical and mental health^25^. Given that individuals of working age spend a large portion of their workday sitting^30^, screen time in this group may be even higher, highlighting the impact of workloads on individuals’ health. Furthermore, individuals with multimorbidity may reduce their physical activity because of illness, suggesting a possible reverse causality, which requires caution in interpreting the results.

Given the negative impact of excessive screen time on the health of the economically active population, especially young people, there is growing interest in interventions that promote active breaks at work. Short-duration physical activities can alleviate pain and discomfort during the workday^25^. In addition, it is necessary to encourage lifestyle changes, such as increased daily physical activity, engaging in moderate-to-vigorous physical activity, and reducing leisure-time screen time. These actions are associated with a lower risk of mortality^30^, resulting in a more active workforce with greater longevity^25,30^.

In this study, the employed population that rated their health negatively had twice the prevalence of multimorbidity. Negative health assessments reflect greater physical and social demands, which contribute to the onset of CNCDs. The disabilities resulting from multimorbidity and awareness of a chronic disease diagnosis also contribute to a poorer health assessment^9^.

It was observed that those who had seen a doctor in the past year and those with health insurance had a positive association with multimorbidity. This condition leads to greater use of health services and an increase in diagnoses^10,22^. Therefore, indicators of service utilization can reveal social inequalities, informing the planning of health interventions^7^. Having health insurance is also related to socioeconomic conditions and may result in greater access to services and a higher likelihood of diagnosis^9^.

The strengths of this study include a robust sample of the employed population across all regions of Brazil, both urban and rural, which ensures external validity. The focus on a specific population allows for the targeting of health policies aimed at promoting and preserving the health of these individuals. Limitations include the reliance solely on self-reported medical diagnoses, which may mask certain inequalities and is subject to classification and surveillance bias. However, in population-based studies, self-reported information is considered valid and robust for monitoring health status^9^. To minimize errors, the PNS standardizes and trains interviewers, ensuring data quality^13^. Finally, the cross-sectional design assesses exposure and outcome simultaneously, precluding causal inference; therefore, some observed associations may be due to reverse causality.

The prevalence of multimorbidity observed in this study was high, with significant associated factors, including habits, behaviors, and social determinants such as educational attainment, place of residence, use of health services, and health insurance coverage. Comprehensive, intersectoral public policies targeting these determinants can prevent CNCDs among employed individuals, improve productivity, and reduce public health costs.

To achieve the goals of the Strategic Action Plan to Tackle NCDs (2021–2030), it is essential to include employed individuals in ongoing monitoring through specific indicators. These data can contribute to proposing changes in production processes, work management, and social contexts, thereby promoting healthier conditions^8^.

The findings of this study can guide health promotion initiatives in various settings—not only in the workplace but also in community and educational settings and in primary care. Strategies such as encouraging physical activity, reducing alcohol consumption, conducting educational campaigns, and expanding access to health services are essential for preventing premature illness. Managers and public policy makers should use this evidence to create healthier and more equitable environments for the working-age population.
