# Supplementary material for: Multimorbidity in the working-age population in Brazil: sociodemographic and behavioral factors
Source: Rev Saude Publica. 2026 Aug 7;60:e36. doi: 10.11606/s1518-8787.2026060007251 (PMC13480949; doi:10.11606/s1518-8787.2026060007251)
Supplement: Translation

# Translation: Multimorbidade na população ocupada no Brasil: fatores sociodemográficos e comportamentais

**Authors:** Tamyres Araújo Andrade Donato, Amanda Cristina de Souza Andrade, Vanessa Moraes Bezerra

**Affiliations:** I Brasil Universidade Federal da Bahia. Instituto Multidisciplinar em Saúde. Vitória da Conquista, BA, Brasil; II Brasil Universidade Estadual do Sudoeste da Bahia. Departamento de Ciências da Saúde. Vitória da Conquista, BA, Brasil; III Brasil Fundação Oswaldo Cruz. Instituto René Rachou. Belo Horizonte, MG, Brasil; IV Brasil Universidade Federal do Mato Grosso. Instituto de Saúde Coletiva. Programa de Pós-Graduação em Saúde Coletiva. Cuiabá, MT, Brasil

**Keywords:** Multimorbidade, População Economicamente Ativa, Brasil, Inquérito de Saúde, Estudos Epidemiológicos

## Abstract

**OBJETIVO:**

Avaliar a prevalência da multimorbidade na população ocupada brasileira e analisar seus determinantes, com ênfase em aspectos sociodemográficos e comportamentais.

**MÉTODOS:**

Realizou-se estudo transversal, utilizando dados da Pesquisa Nacional de Saúde 2019, com indivíduos ocupados de 18 anos ou mais. O desfecho foi a multimorbidade, caracterizada pela presença simultânea de duas ou mais doenças crônicas não transmissíveis, a partir de uma lista de 14 morbidades. Para avaliar a associação entre as variáveis sociodemográficas, hábitos e comportamentos e de saúde/serviço foi utilizada a regressão de Poisson com variância robusta.

**RESULTADOS:**

Dentre os 52.475 indivíduos ocupados, 22,6% (IC95% 21,9–23,2) apresentaram multimorbidade, sendo mais prevalente em: mulheres; aqueles com 30 anos ou mais; menor escolaridade; raça/cor autodeclarada como branca e em residentes da zona urbana. Também apresentaram associação positiva com multimorbidade a participação em programas públicos para prática de atividade física, o consumo excessivo episódico de álcool, o tempo de tela no lazer, a autoavaliação da saúde como regular/ruim ou muito ruim, consulta médica no último ano e possuir plano de saúde.

**CONCLUSÃO:**

Os achados evidenciam a ocorrência significativa de multimorbidade na população ocupada no Brasil, com destaque para determinantes sociais e comportamentais. Tais resultados reforçam a importância de estratégias de promoção da saúde em múltiplos contextos, incluindo ambientes de trabalho, comunitários e de atenção primária.

## INTRODUÇÃO

As doenças crônicas não transmissíveis (DCNT) afetam principalmente populações mais expostas a fatores de risco, como socioeconômicos e de estilo de vida ou com acesso limitado a informações e serviços de saúde. O aumento da prevalência das DCNT e sua coexistência em um mesmo indivíduo resultam em pior qualidade de vida, limitações, incapacidades e alta mortalidade prematura^1^, configurando-se como um problema de saúde pública.

A multimorbidade é definida como a coexistência de duas ou mais doenças crônicas em um mesmo indivíduo^1^. As estimativas de prevalência variam amplamente conforme o contexto e os critérios adotados. Em estudo internacional com adultos mais velhos, a prevalência foi de 55,1%^2^. No Brasil, investigação de base populacional com adultos de 20 a 59 anos estimou prevalência de 10,9%, a partir de diagnóstico médico autorreferido^3^. Entre a população ocupada, observa-se marcante heterogeneidade: prevalência de 0,9% em trabalhadores europeus^1^ e valores mais elevados no Brasil, variando de 28,6% entre trabalhadores urbanos a 41,5% entre trabalhadores rurais^4,5^. Esses achados evidenciam a influência do contexto ocupacional e das desigualdades sociais na ocorrência da multimorbidade.

Atualmente, a multimorbidade destaca-se como importante causa de incapacidade entre indivíduos ocupados, elevando o risco de acidentes de trabalho, reduzindo o desempenho laboral e aumentando o absenteísmo^5^. Além dos prejuízos físicos, a multimorbidade eleva a demanda por serviços de saúde, incluindo consultas, hospitalizações e serviços de emergência. Este cenário pode gerar impactos econômicos relevantes, devido à redução da capacidade produtiva, afastamentos prolongados e maior uso de serviços assistenciais e previdenciários^6^.

Diversos aspectos vivenciados pela população ocupada influenciam o desenvolvimento da multimorbidade, incluindo instabilidade laboral, baixos salários, múltiplos vínculos e pressões por produtividade, que atuam como estressores crônicos^6^. Para além do estresse, a própria atividade laboral e o ambiente de trabalho podem constituir fatores de risco à saúde, como exposições físicas, químicas e ergonômicas, já associadas a condições cardiovasculares, musculoesqueléticas e neoplásicas^4,5^. Adicionalmente, jornadas extensas, trabalho em turnos, tempo prolongado de deslocamento e rotinas rígidas dificultam a adoção de comportamentos saudáveis, como a prática regular de atividade física^5^. Essas condições também limitam o acesso oportuno aos serviços de saúde, comprometendo ações de promoção, prevenção, diagnóstico precoce e acompanhamento das condições crônicas^7^.

Nesse cenário, o Ministério da Saúde lançou, em 2021, o Plano de Ações Estratégicas para o Enfrentamento das Doenças Crônicas e Agravos não Transmissíveis no Brasil (2021–2030), que propõe preveni-las por meio de mudanças nos processos produtivos, estratégias de promoção da saúde e intervenções nos fatores de risco ocupacionais^8^. A investigação da multimorbidade entre indivíduos ocupados no Brasil pode gerar evidências para subsidiar gestores, profissionais de saúde e empregadores na formulação de políticas que contribuam para sua prevenção^5^.

Estudos com dados da Pesquisa Nacional de Saúde (PNS) investigaram a multimorbidade em diferentes segmentos da população brasileira, incluindo população geral^9^, idosos^10^, jovens^11^, bem como subgrupos ocupacionais específicos, como trabalhadores agrícolas^7^. Contudo, poucos a abordaram especificamente entre pessoas ocupadas, que representam a população economicamente ativa e produtiva do País. Considerando que a multimorbidade pode surgir precocemente, especialmente em contextos de desigualdade social^3^, é necessário compreender os fatores associados nessa população para subsidiar políticas de promoção da saúde, prevenção e planejamento intersetorial.

Este estudo objetivou avaliar a prevalência de multimorbidade na população ocupada brasileira e analisar os aspectos sociodemográficos e comportamentais associados.

## MÉTODOS

### Desenho do Estudo

Estudo transversal com dados da PNS, inquérito nacional epidemiológico de base domiciliar, realizado em 2019 pelo Instituto Brasileiro de Geografia e Estatística (IBGE) em parceria com Ministério da Saúde. As informações, coletadas por entrevistas domiciliares, incluíram características socioeconômicas, presença de doenças e estilo de vida^12^. Foram visitados 94.114 domicílios e realizadas 90.846 entrevistas^13^. Destas, 52.475 pessoas com 18 anos ou mais referiram estar ocupados ou trabalhando na semana de referência (21 a 27 de julho de 2019)^14^, compondo a amostra deste estudo.

### Desfecho

O desfecho foi a multimorbidade, definida como presença simultânea de duas ou mais DCNT^3,11^, avaliada a partir de 14 morbidades: hipertensão arterial, diabetes, hipercolesterolemia, problemas cardíacos (infarto, angina ou insuficiência cardíaca), acidente vascular cerebral, asma, artrite ou reumatismo, problema crônico de coluna (dor crônica nas costas ou no pescoço, lombalgia, dor ciática, problemas nas vértebras ou disco), distúrbios musculoesqueléticos relacionados ao trabalho, depressão, outras doenças mentais (transtorno de ansiedade, síndrome do pânico, esquizofrenia, transtorno bipolar, psicose ou transtorno obsessivo compulsivo), doenças pulmonares (enfisema pulmonar, bronquite crônica ou doença pulmonar obstrutiva crônica), câncer e doença renal crônica. Foram identificadas pela pergunta: “Algum médico já lhe deu o diagnóstico de (nome da doença)”^12^. A variável multimorbidade foi dicotomizada em: “Sim”, presença de duas ou mais DCNT; “Não”, ausência ou apenas uma DCNT^10^.

### Variáveis Independentes

As variáveis independentes foram escolhidas de acordo revisão prévia da literatura^10,11,15,16^, sendo divididas nos seguintes blocos: sociodemográfico, hábitos e comportamentos, e saúde/serviço. No bloco sociodemográfico, incluíram-se: sexo (masculino, feminino); idade (18–29, 30–39, 40–59, ≥ 60 anos); escolaridade (sem instrução à médio incompleto, médio completo à superior completo); raça/cor autodeclarada (branca, preta/parda). A proporção de indivíduos autodeclarados indígenas (702; 0,75%) e amarelos (692; 0,74%) foi baixa na amostra da PNS. Considerando o número reduzido de observações e a possibilidade de estimativas imprecisas, optou-se por restringir a análise às categorias branca e preta/parda. Regime de trabalho por turno de 24 horas (sim, não); trabalho noturno (sim, não) e local de moradia (urbano, rural).

No bloco de hábitos e comportamentos, considerou-se: participação em programa público para prática de atividade física (sim, não conhece/não participa); consumo excessivo episódico de álcool (consumo de cinco ou mais doses de bebida alcoólica em uma única ocasião para homens e mulheres); tabagismo atual (sim, não); prática de atividade física (classificados como inativos aqueles que praticaram menos do que 150 minutos/semana, nos domínios do lazer, trabalho e deslocamento para o trabalho)^15^. A prática de atividade física em grupo (prática de atividades esportivas, exercícios físicos, recreativos ou artísticos) foi dicotomizada em “mais de uma vez na semana a três vezes no mês” e “Não realiza/Apenas algumas vezes no ano”. Foram classificados com tempo excessivo de tela aqueles que passaram 3 horas ou mais por dia assistindo TV ou utilizando computador, tablet ou celular para lazer (excluindo o trabalho)^16^.

No bloco de saúde/serviço, considerou-se: autoavaliação de saúde (muito boa/boa, regular/ruim/muito ruim)^10^; consulta médica no último ano (sim: até um ano, não: mais de um ano/nunca foi)^11^ e posse de plano de saúde (sim, não)^10^.

### Análise Estatística

Realizou-se análise descritiva e estimativa da prevalência de multimorbidade e intervalo de confiança de 95% (IC95%). Para verificar os fatores associados, foram realizadas análises bivariada e múltipla com estimativas das razões de prevalência (RP) e IC95% por meio da regressão de Poisson com variância robusta. A análise múltipla seguiu uma estratégia de entrada hierárquica por blocos de variáveis^17^. Inicialmente, foi realizada análise bivariada, e aquelas variáveis que apresentaram p-valor < 0,20 foram elegíveis para inclusão na análise múltipla. Esse critério foi adotado para evitar a exclusão precoce de potenciais fatores de confusão. Na análise múltipla com entrada hierárquica, as variáveis foram inseridas em blocos conforme o modelo proposto (Figura). Para cada bloco (sociodemográfico, hábitos e comportamentos e saúde/serviço) foi ajustado um modelo múltiplo, excluindo sucessivamente as variáveis com maior p-valor, até que permanecesse em cada bloco aquelas com p-valor ≤ 0,05 após o ajuste pelas variáveis do mesmo bloco e dos blocos hierarquicamente superiores.

**Figura:** Modelo hierarquizado para análise de dados.

O critério de Akaike (AIC) foi utilizado na comparação de modelos, sendo considerado o melhor modelo aquele com menor AIC. As análises foram feitas no Stata (versão 16), modo *survey*, que considera o desenho complexo da amostra.

### Aspectos Éticos

A PNS foi aprovada pela Comissão Nacional de Ética em Pesquisa (parecer nº 3.529.376).

## RESULTADOS

Dos 52.475 indivíduos ocupados, 11.638 (22,6%; IC95% 21,9–23,2) apresentaram multimorbidade. As DCNT mais prevalentes (Tabela 1) foram hipertensão arterial (19,3%), problema crônico de coluna (19,0%), hipercolesterolemia (13,2%), depressão (7,7%) e diabetes (5,4%).

**Tabela 1:** Prevalência de doenças crônicas não transmissíveis autorreferidas na população ocupada brasileira, Pesquisa Nacional de Saúde, 2019 (n = 52.475).

Doenças crônicas não transmissíveis autorreferidas	Prevalência (%)	IC95%
Hipertensão arterial	19,3	19,0–19,7
Problema crônico de coluna	19,0	18,6–19,3
Hipercolesterolemia	13,2	12,8–13,4
Depressão	7,7	7,4–7,9
Diabetes mellitus	5,4	5,2–5,6
Outras doenças mentais	5,1	4,9–5,2
Artrite ou reumatismo	4,9	4,8–5,1
Asma	4,8	4,6–5,0
Doenças cardiovasculares	3,2	3,0–3,3
Doenças reumáticas relacionadas ao trabalho	2,2	2,1–2,4
Câncer	1,6	1,5–1,7
Doença renal crônica	1,1	1,0–1,2
Acidente vascular cerebral	1,0	0,9–1,1
Doenças pulmonares	1,0	0,9–1,1
Multimorbidade	22,6	21,9–23,2

IC95%: intervalo de confiança de 95%.

Em relação às características da amostra (Tabela 2), foi observado maior percentual de homens (55,5%), idade de 40 a 59 anos (41,1%), escolaridade entre ensino médio completo à superior completo (59,8%) e raça/cor preta ou parda (55,3%). Quanto às características ocupacionais, 1,7% faziam regime de turno de 24 horas e 13,5% turnos noturnos. Um maior percentual dos participantes residia em zona urbana (87,7%). Quanto aos hábitos e comportamentos, a maioria não participava de programas públicos para prática de atividade física, 22,1% relataram consumir cinco ou mais doses de álcool em uma única ocasião, nos últimos 30 dias, 12,9% consumiam tabaco, 27,6% eram inativos fisicamente, 59,5% informaram não realizar atividades físicas em grupo ou realizá-las apenas algumas vezes no último ano e 35% passavam três ou mais horas do dia sentados em frente às telas. Para as variáveis de saúde, 26,2% avaliaram sua saúde como regular, ruim ou muito ruim, 21,9% não consultaram médico no último ano e 69,7% não tinham plano de saúde.

**Tabela 2:** Características da população ocupada brasileira, Pesquisa Nacional de Saúde, 2019 (n = 52.475).

Variáveis	Prevalência (%)	IC95%
**Sociodemográficas**		
Sexo		
Masculino	55,5	54,7–56,4
Feminino	44,5	43,6–45,3
Idade		
18–29 anos	23,2	22,4–24,0
30–39 anos	26,9	26,3–27,7
40–59 anos	41,1	40,3–41,8
≥ 60 anos	8,8	8,4–9,2
Escolaridade		
Ensino médio completo à ensino superior completo	59,8	59,0–60,7
Sem instrução à ensino médio incompleto	40,2	39,3–41,0
Raça/cor		
Pretos/pardos	55,3	54,4–56,2
Brancos	44,7	43,8–45,6
Regime de trabalho por turno de 24 horas		
Sim	1,7	1,5–1,9
Não	98,3	98,2–98,5
Trabalho noturno		
Sim	13,5	12,9–14,1
Não	86,5	85,9–87,1
Local de moradia		
Rural	12,3	11,9–12,8
Urbano	87,7	87,2–88,1
**Hábitos e comportamentos**		
Participação em programa público para prática de atividade física		
Não participa/não conhece	97,7	97,4–97,9
Sim	2,3	2,1–2,6
Consumo excessivo episódico de álcool		
Sim	22,1	21,4–22,8
Não	77,9	77,2–78,6
Consumo de tabaco atualmente		
Sim	12,9	12,4–13,5
Não	87,1	86,5–87,6
Prática de atividade física		
Ativo	72,4	71,6–73,2
Inativo	27,6	26,8–28,3
Prática de atividade física em grupo no último ano		
> Uma vez por semana a três vezes por mês	40,5	39,7–41,4
Não realiza/apenas algumas vezes no ano	59,5	59,5–58,6
Tempo de tela no lazer		
≥ 3 horas	35,0	34,1–35,8
< 3 horas	65,0	64,2–65,9
**Saúde/serviço**		
Autoavaliação de saúde		
Muito boa/boa	73,8	73,2–74,5
Regular/ruim/muito ruim	26,2	25,5–26,9
Consulta ao médico no último ano		
Não	21,9	21,3–22,5
Sim	78,1	77,5–78,8
Posse de plano de saúde		
Não	69,7	68,7–70,6
Sim	30,3	29,4–31,2

IC95%: intervalo de confiança de 95%.

Na análise bivariada, a multimorbidade apresentou associação estatisticamente significativa com variáveis sociodemográficas, comportamentais e relacionadas à saúde, incluindo sexo, idade, escolaridade, raça/cor, local de moradia, participação em programa público para a prática de atividade física, consumo excessivo episódico de álcool, atividade física, prática de atividade física em grupo, tempo de tela no lazer, autoavaliação de saúde, consulta ao médico no último ano e posse de plano de saúde (Tabela 3). As associações observadas variaram quanto à direção e magnitude, sendo exploradas na análise ajustada subsequente.

**Tabela 3:** Prevalência e razão de prevalência bruta da multimorbidade segundo características sociodemográficas, hábitos e comportamentos e condições de saúde/serviços na população ocupada brasileira, Pesquisa Nacional de Saúde, 2019.

Variáveis	Prevalência (%) (IC95%)	RP (IC95%)	p-valor
**Sociodemográficas**			
Sexo			
Masculino	17,4 (16,6–18,2)	1,00	< 0,001
Feminino	29,0 (27,9–30,1)	1,67 (1,57–1,76)	
Idade			
18–29 anos	8,9 (7,9–9,9)	1,00	< 0,001
30–39 anos	14,7 (13,7–15,8)	1,66 (1,45–1,90)	
40–59 anos	30,2 (29,1–31,3)	3,41 (3,03–3,83)	
≥ 60 anos	46,8 (44,5–49,1)	5,28 (4,68–5,96)	
Escolaridade			
Médio completo à superior completo	20,6 (19,7–21,5)	1,00	< 0,001
Sem instrução à médio incompleto	25,5 (24,4–26,6)	1,23 (1,17–1,31)	
Raça/cor			
Pretos/pardos	20,9 (20,0–21,7)	1,00	< 0,001
Brancos	24,6 (23,6–25,7)	1,18 (1,12–1,25)	
Regime de trabalho por turno de 24 horas			
Sim	19,0 (15,5–23,0)	1,00	0,083
Não	22,6 (21,9–23,2)	1,19 (0,98–1,45)	
Trabalho noturno			
Sim	22,2 (20,5–24,0)	1,00	0,691
Não	22,6 (21,9–23,3)	1,01 (0,93–1,11)	
Local de moradia			
Rural	19,6 (18,4–20,9)	1,00	< 0,001
Urbano	23,0 (22,2–23,7)	1,17 (1,08–1,26)	
**Hábitos e comportamentos**			
Participação em programa público para prática de atividade física			
Não participa /não conhece	22,3 (21,7–23,0)	1,00	< 0,001
Sim	31,5 (26,9–36,5)	1,41 (1,20–1,64)	
Consumo excessivo episódico de álcool			
Não	23,6 (22,8–24,4)	1,00	< 0,001
Sim	19,0 (17,9–20,2)	0,80 (0,75–0,86)	
Consumo de tabaco atualmente			
Não	22,6 (21,8–23,3)	1,00	0,863
Sim	22,4 (20,7–24,2)	0,99 (0,91–1,07)	
Prática de atividade física			
Ativo	21,6 (20,8–22,3)	1,00	< 0,001
Inativo	25,1 (23,9–26,4)	1,17 (1,09–1,24)	
Prática de atividade física em grupo no último ano			
> Uma vez por semana a três vezes por mês	19,8 (18,8–20,9)	1,00	< 0,001
Não realiza/apenas algumas vezes no ano	24,4 (23,6–25,3)	1,23 (1,16–1,31)	
Tempo de tela no lazer			
< 3 horas	23,3 (22,4–24,1)	1,00	0,002
≥ 3 horas	21,2 (20,2–22,3)	0,91 (0,86–0,96)	
**Saúde/serviço**			
Autoavaliação de saúde			
Muito boa/boa	16,0 (15,3–16,7)	1,00	< 0,001
Regular/ruim/muito ruim	41,1 (39,7–42,5)	2,57 (2,44–2,71)	
Consulta ao médico no último ano			
Não	8,0 (7,2–8,8)	1,00	< 0,001
Sim	26,6 (25,8–27,5)	3,34 (3,01–3,70)	
Posse de plano de saúde			
Não	20,8 (20,1–21,5)	1,00	< 0,001
Sim	26,6 (25,3–28,0)	1,28 (1,20–1,36)	

RP: razão de prevalência; IC95%: intervalo de confiança de 95%.

Na análise múltipla por regressão de Poisson (Tabela 4), a prevalência de multimorbidade foi maior entre mulheres (RP = 1,67; IC95% 1,59–1,77), e apresentou um gradiente dose-resposta com a idade, ou seja, quanto mais elevada a idade, maior a prevalência de multimorbidade (RP = 5,14; IC95% 4,54–5,83). Indivíduos ocupados com menor escolaridade (RP = 1,08; IC95% 1,02–1,15), os brancos (RP = 1,11; IC95% 1,05–1,17) e moradores urbanos (RP = 1,11; IC95% 1,04–1,19) apresentaram maior prevalência do desfecho. Também apresentaram associação positiva com a multimorbidade a participação em programas públicos para prática de atividade física (RP = 1,26; IC95% 1,09–1,44), o consumo excessivo episódico de álcool (RP = 1,08; IC95% 1,01–1,15) e o tempo de tela no lazer (RP = 1,13; IC95% 1,07–1,19). Para variáveis de saúde, foi mantida a associação positiva com a multimorbidade a autoavaliação negativa da saúde (RP = 2,12; IC95% 2,01–2,23), consulta médica no último ano (RP = 2,45; IC95% 2,20–2,72) e posse de plano de saúde (RP = 1,26; IC95% 1,19–1,33).

**Tabela 4:** Análise múltipla por regressão de Poisson dos fatores associados a multimorbidade na população ocupada brasileira, Pesquisa Nacional de Saúde, 2019.

Variáveis	Modelo 1^a^		Modelo 2^b^		Modelo 3^c^	
	RP	IC95%	RP	IC95%	RP	IC95%
**Sociodemográficas**						
Sexo						
Masculino	1,00	-	1,00	-	1,00	-
Feminino	1,67	1,59–1,77	1,69	1,60–1,79	1,48	1,40–1,56
Idade						
18–29 anos	1,00	-	1,00	-	1,00	-
30–39 anos	1,65	1,45–1,89	1,70	1,48–1,94	1,60	1,40–1,82
40–59 anos	3,30	2,93–3,73	3,44	3,05–3,87	2,91	2,59–3,28
≥ 60 anos	5,14	4,54–5,83	5,36	4,73–6,08	4,09	3,61–4,64
Escolaridade						
Ensino médio completo à ensino superior completo	1,00	-	1,00	-	1,00	-
Sem instrução à ensino médio incompleto	1,08	1,02–1,15	1,09	1,03–1,16	1,06	0,99–1,13
Raça/cor						
Pretos/pardos	1,00	-	1,00	-	1,00	-
Brancos	1,11	1,05–1,17	1,11	1,06–1,18	1,15	1,09–1,21
Local de moradia						
Rural	1,00	-	1,00	-	1,00	-
Urbano	1,11	1,04–1,19	1,08	1,01–1,16	1,05	0,98–1,12
**Hábitos e comportamentos**						
Participação em programa público para prática de atividade física						
Não participa /não conhece			1,00	-	1,00	-
Sim			1,26	1,09–1,44	1,26	1,11–1,44
Consumo excessivo episódico de álcool						
Não			1,00	-	1,00	-
Sim			1,08	1,01–1,15	1,11	1,04–1,19
Tempo de tela no lazer						
< 3 horas			1,00	-	1,00	-
≥ 3 horas			1,13	1,07–1,19	1,13	1,07–1,19
**Saúde/serviço**						
Autoavaliação de saúde						
Muito boa/boa					1,00	-
Regular/ruim/muito ruim					2,12	2,01–2,23
Consulta ao médico no último ano						
> Um ano ou nunca foi					1,00	-
Até um ano					2,45	2,20–2,72
Posse de plano de saúde						
Não					1,00	-
Sim					1,26	1,19–1,33
Critério Akaike^d^	4025063		4021596		3801403	

RP: razão de prevalência; IC95%: intervalo de confiança de 95%.

^a^Modelo 1: ajustado pelas variáveis sociodemográficas.

^b^Modelo 2: ajustado pelas variáveis sociodemográficas e de hábitos e comportamentos.

^c^Modelo 3: ajustado pelas variáveis sociodemográficas, hábitos e comportamentos e saúde/serviço.

^d^ Ponderado pelo peso amostral.

## DISCUSSÃO

Este estudo evidenciou elevada prevalência de multimorbidade na população brasileira em idade produtiva, especialmente entre mulheres e indivíduos de maior idade. Observou-se também associação com condições socioeconômicas e contextuais, como menor escolaridade e residência em áreas urbanas. Além disso, a multimorbidade esteve relacionada a comportamentos de risco e a maior utilização de serviços de saúde, sugerindo que sua ocorrência resulta da interação entre fatores sociodemográficos, comportamentais e de acesso aos cuidados.

As doenças mais prevalentes neste estudo, como hipertensão arterial, problema crônico de coluna, hipercolesterolemia, depressão, diabetes e outras doenças mentais são consistentes com o perfil de multimorbidade descrito na literatura com população geral e de trabalhadores^3,6,11^. Atualmente, no cenário brasileiro, condições do sistema osteomuscular e do tecido conjuntivo (CID-10 M) e transtornos mentais e comportamentais (CID-10 F) figuram entre os principais motivos de afastamento laboral tanto acidentários quanto não acidentários^18^. Essas condições apresentam forte associação com ambientes ocupacionais adversos, caracterizados por jornadas prolongadas, instabilidade no emprego e elevada pressão por produtividade, o que reforça o papel do trabalho no desencadeamento e agravamento de condições crônicas com repercussões físicas e mentais^5^. Como consequência, tais agravos impactam negativamente a produtividade, com aumento do absenteísmo, da invalidez e da aposentadoria precoce, além de impor ônus significativos ao sistema de saúde e previdenciário^1^.

Neste estudo, a prevalência de multimorbidade foi maior entre mulheres em comparação aos homens, resultado já consolidado em literatura^5,9^. A maior utilização de serviços de saúde pelas mulheres possibilita maior acesso a diagnósticos médicos^9^. Além disso, a dupla jornada, que combina trabalho profissional e responsabilidades domésticas e familiares, aumenta a exposição a estresse e pode contribuir para o desenvolvimento de doenças crônicas^19^.

A multimorbidade foi mais prevalente entre indivíduos ocupados com 30 anos ou mais, indicando ocorrência de múltiplas condições crônicas em grupos economicamente ativos. A população em idade produtiva está exposta a comportamentos de risco à saúde e a diversas situações de estresse, favorecendo o acúmulo de doenças ao longo do tempo^6,19^. Esse achado reforça a originalidade da análise ao evidenciar que doenças crônicas podem surgir precocemente, possivelmente deflagradas por contextos sociais e hábitos de vida, antes mesmo de longa trajetória ocupacional^3,11^. Essa antecipação do adoecimento traz implicações para saúde e economia, pois indivíduos em idades produtivas, com multimorbidade, podem enfrentar barreiras à inserção e permanência no mercado de trabalho, maior risco de demissões, aposentadoria precoce e recebimento de benefícios por incapacidade^1,6^. A esses grupos vulneráveis, são necessárias políticas e programas específicos^7^ para reduzir multimorbidade, absenteísmo e custos com DCNT^1^. A prevenção e o cuidado à saúde da população ocupada devem ser vistos como investimentos estratégicos para o desenvolvimento econômico.

Menor escolaridade associou-se positivamente à multimorbidade, achado também observado em outros grupos populacionais^1^. A escolaridade expressa posição socioeconômica e influencia trajetórias ocupacionais, exposição a condições laborais precárias e acesso a recursos de proteção à saúde. Contextos de vulnerabilidade, caracterizados por vínculos empregatícios instáveis, sobrecarga psicossocial e menor acesso a serviços de saúde favorecem o surgimento precoce e o controle inadequado de condições crônicas^20,21^. Ademais, o contexto de moradia constitui importante determinante social da saúde. Populações residentes em periferias urbanas socialmente negligenciadas enfrentam maior privação socioeconômica, menor acesso à infraestrutura urbana e aos serviços públicos e maior exposição a fatores ambientais adversos, contribuindo para o agravamento de doenças crônicas e para a ocorrência de multimorbidade em idades mais precoces^20,21^.

Neste estudo, observou-se maior prevalência de multimorbidade entre indivíduos brancos. Contudo, é importante considerar que o desfecho foi baseado no autorrelato de diagnóstico médico, o que pode refletir diferenças no acesso e na utilização dos serviços de saúde. Pessoas brancas, geralmente, apresentam maior escolaridade e renda e, consequentemente, maior probabilidade de receber diagnóstico oportuno de doenças crônicas, o que pode superestimar a prevalência nesse grupo quando comparada à população negra^22^. Evidências recentes, tanto internacionais quanto nacionais, têm demonstrado maior carga de multimorbidade entre indivíduos pretos e pardos, especialmente quando considerados marcadores estruturais de desigualdade^22,23^. Essas desigualdades são produzidas ao longo do curso de vida e incluem maior exposição a condições socioeconômicas adversas, segregação territorial e experiências de discriminação racial, fatores associados ao adoecimento crônico acumulado^23^. Assim, estudos baseados exclusivamente em autorrelato de diagnóstico podem subestimar iniquidades raciais em saúde, reforçando a necessidade de análises que incorporem determinantes estruturais e contextuais na compreensão da multimorbidade^22^.

Indivíduos ocupados residentes em áreas urbanas apresentaram maior prevalência de multimorbidade, resultado semelhante ao encontrado em um estudo desenvolvido na Colômbia^23^. A maior oferta de serviços de saúde nesses locais favorece o diagnóstico de doenças^10,19^. Ambientes urbanos também expõem os indivíduos a estressores, como trânsito, violência e poluição, que podem contribuir para multimorbidade^7^. Já nas zonas rurais, barreiras geográficas, transporte precário e maior pobreza dificultam o acesso aos serviços, gerando menores oportunidades de cuidado^7^. Nesta pesquisa, como o diagnóstico foi autorrelatado, aqueles com maior acesso aos serviços de saúde tiveram mais chances de relatar doenças.

Quanto aos hábitos e comportamentos, a participação em programas públicos para prática de atividade física foi maior entre indivíduos ocupados com multimorbidade. Pessoas com DCNT frequentam mais os serviços de saúde e recebem mais informações sobre essas iniciativas^24^. O acesso a espaços públicos que incentivem hábitos saudáveis é essencial para prevenir DCNT, reduzindo comportamentos de risco à saúde, o que estimula um estilo de vida mais ativo^11^. A estratégia do Ministério da Saúde para controle dessas doenças prioriza o envelhecimento ativo^8^. Como muitos indivíduos em idade produtiva enfrentam barreiras para a prática regular de atividades físicas, é importante que as atividades públicas sejam acessíveis e oferecidas em horários alternativos^24^. Também são necessárias intervenções nas políticas de saúde e nos ambientes laborais, promovendo atividades leves e de curta duração, como caminhadas e o uso de escadas, incorporadas à rotina do trabalho^25^.

Neste estudo, indivíduos ocupados que relataram consumir cinco ou mais doses de álcool em uma única ocasião, nos últimos trinta dias, apresentaram maior prevalência de multimorbidade. Esse achado é particularmente relevante entre indivíduos mais jovens, visto que o uso abusivo de bebidas alcoólicas pode antecipar o surgimento de doenças crônicas em idades produtivas, o que reforça a necessidade de intervenções precoces^26^. Por se tratar de um fator de risco modificável, é fundamental investir em políticas públicas de prevenção e intervenção específicas para esta população.

O consumo excessivo episódico de álcool pode estar relacionado a condições específicas do trabalho, como longas jornadas, instabilidade, atividades desvalorizadas e esforço físico ou mental elevados^26^. Fatores contextuais, sociodemográficos e estilo de vida também podem contribuir para esse comportamento^27^. Esse padrão de consumo afeta negativamente as esferas ocupacional, familiar e social^26^. Como estratégias de enfrentamento, recomenda-se ampliar e qualificar o rastreamento e diagnóstico do consumo excessivo de álcool no trabalho e lazer, garantindo acesso a serviços de reabilitação psicossocial quando necessário^28^. Adicionalmente, ações intersetoriais envolvendo órgãos reguladores e de saúde são essenciais para reduzir o consumo, priorizando grupos vulneráveis^27^.

O tempo de tela, desconsiderando o tempo sentado no trabalho, associou-se positivamente à multimorbidade, especialmente em adultos ocupados jovens (18 a 39 anos). O aumento do tempo dedicado ao lazer passivo, como o uso telas, pode explicar esse achado^29^. Jornadas excessivas e tipo de trabalho desempenhado também favorecem o comportamento sedentário^30^, que contribui para outros riscos, podendo gerar impactos negativos na saúde física e mental^25^. Considerando que indivíduos em idade produtiva passam grande parte da jornada ocupacional sentados^30^, o tempo de tela nesse grupo pode ser ainda maior, evidenciando o impacto das cargas de trabalho sobre a saúde dos indivíduos. Além disso, indivíduos com multimorbidade podem reduzir atividades físicas em decorrência do adoecimento, caracterizando possível causalidade reversa, o que exige cautela na interpretação dos resultados.

Dado o impacto negativo do tempo excessivo de tela na saúde dos economicamente ativos, especialmente jovens, cresce o interesse por intervenções que promovam pausas ativas no trabalho. Atividades físicas de curta duração podem aliviar dores e desconfortos durante a jornada^25^. Além disso, é necessário incentivar mudanças no estilo de vida, como maior mobilidade diária, realização de atividades físicas moderadas a vigorosas e redução do tempo de tela no lazer. Essas ações estão associadas a menor risco de mortalidade^30^, formando uma força de trabalho mais ativa e com maior longevidade^25,30^.

Neste estudo, a população ocupada que avaliou negativamente sua saúde, apresentou o dobro da prevalência de multimorbidade. Avaliações negativas refletem maiores demandas físicas e sociais, que contribuem para o surgimento de DCNT. As incapacidades, provenientes da multimorbidade, e o conhecimento sobre algum diagnóstico de doença crônica também determinam uma pior avaliação da saúde^9^.

Foi observado que aqueles que consultaram um médico no último ano e os que possuíam plano de saúde apresentaram associação positiva com multimorbidade. Essa condição leva a maior uso dos serviços de saúde e aumento no diagnóstico^10,22^. Por isso, indicadores de utilização de serviços podem revelar desigualdades sociais, subsidiando o planejamento de ações em saúde^7^. Ter plano de saúde também está relacionado às condições socioeconômicas e pode determinar maior acesso aos serviços e maiores chances de diagnóstico^9^.

Os pontos fortes deste estudo incluem a amostra robusta da população ocupada em todas as regiões do Brasil, urbana e rural, conferindo validade externa. O foco em população específica permite direcionar políticas de saúde para valorização e preservação da saúde desses indivíduos. Como limitações, tem-se a utilização apenas do autorrelato de diagnóstico médico, o que pode mascarar algumas desigualdades, estando sujeito ao viés de classificação e vigilância. Contudo, em estudos populacionais, informações autorreferidas são consideradas válidas e robustas para monitoramento da situação de saúde^9^. A fim de minimizar erros, a PNS padroniza e treina entrevistadores, assegurando a qualidade dos dados^13^. Por fim, o desenho transversal avalia exposição e desfecho simultaneamente, não permitindo inferência causal, logo, algumas associações observadas podem ser devido à causalidade reversa.

A prevalência de multimorbidade observada neste estudo foi elevada, com fatores associados importantes, incluindo hábitos, comportamentos e determinantes sociais como escolaridade, local de residência, uso dos serviços de saúde e posse de plano. Políticas públicas abrangentes e intersetoriais voltadas a esses determinantes podem prevenir DCNT entre indivíduos ocupados, melhorar a produtividade e reduzir custos para a saúde pública.

Para alcançar as metas do Plano de Ações Estratégicas para o Enfrentamento das DCNT no Brasil (2021–2030), é fundamental incluir os indivíduos ocupados no monitoramento contínuo por meio de indicadores específicos. Esses dados podem contribuir para a proposição de mudanças nos processos produtivos, na gestão do trabalho e nos contextos sociais, promovendo condições mais saudáveis^8^.

Os achados desse estudo podem orientar ações de promoção da saúde em diversos cenários, não apenas no ambiente de trabalho, mas também em espaços comunitários, educacionais e na atenção primária. Estratégias como incentivo à prática de atividade física, redução do consumo de álcool, campanhas educativas e ampliação do acesso aos serviços de saúde são essenciais para prevenir o adoecimento precoce. Gestores e formuladores de políticas públicas devem usar essas evidências para criar ambientes mais saudáveis e equitativos para a população em idade produtiva.
